# Fas Ligand-mediated cytotoxicity of CD4+ T cells during chronic retrovirus infection

**DOI:** 10.1038/s41598-017-08578-7

**Published:** 2017-08-10

**Authors:** Anna Malyshkina, Elisabeth Littwitz-Salomon, Kathrin Sutter, Gennadiy Zelinskyy, Sonja Windmann, Simone Schimmer, Annette Paschen, Hendrik Streeck, Kim J. Hasenkrug, Ulf Dittmer

**Affiliations:** 1 Institute for Virology, University Hospital Essen, University of Duisburg-Essen, Essen, Germany; 2 Department of Dermatology, Venereology, and Allergology, University Hospital Essen, University of Duisburg-Essen, Essen, Germany; 3 Institute for HIV Research, University Hospital Essen, University Duisburg-Essen, Essen, Germany; 4 Laboratory of Persistent Viral Diseases, Rocky Mountain Laboratories, National Institute of Allergy and Infectious Diseases, National Institutes of Health, Hamilton, Montana USA

## Abstract

CD4+ helper T cells and cytotoxic CD8+ T cells are key players for adaptive immune responses against acute infections with retroviruses. Similar to textbook knowledge the most important function of CD4+ T cells during an acute retrovirus infection seems to be their helper function for other immune cells. Whereas there was no direct anti-viral activity of CD4+ T cells during acute Friend Virus (FV) infection, they were absolutely required for the control of chronic infection. During chronic FV infection a population of activated FV-specific CD4+ T cells did not express cytotoxic molecules, but Fas Ligand that can induce Fas-induced apoptosis in target cells. Using an MHC II-restricted *in vivo* CTL assay we demonstrated that FV-specific CD4+ T cells indeed mediated cytotoxic effects against FV epitope peptide loaded targets. CD4 + CTL killing was also detected in FV-infected granzyme B knockout mice confirming that the exocytosis pathway was not involved. However, killing could be blocked by antibodies against FasL, which identified the Fas/FasL pathway as critical cytotoxic mechanism during chronic FV infection. Interestingly, targeting the co-stimulatory receptor CD137 with an agonistic antibody enhanced CD4+ T cell cytotoxicity. This immunotherapy may be an interesting new approach for the treatment of chronic viral infections.

## Introduction

Viral replication and spread in the acute phase of an infection is usually under the control of CD8+ T cells. This has been described for human and mouse infections such as HIV^1^, LCMV^2^, and Friend virus (FV)^3^. Activated CD8+ T cells express cytotoxic granules that contain perforin and granzymes during acute viral infections^4^. The release of these molecules and subsequent killing of infected cells results in reduction of viral loads. However, during the chronic phase of infection CD8+ T lymphocytes often become functionally exhausted through several mechanisms including suppression by regulatory T cells^5^ and/or sustained expression of inhibitory receptors, such as PD-1^6–8^. CD8+ T cell exhaustion results in decreased killing efficiency *in vivo*, low expression of cytotoxic granules, and reduced cytokine production. Such a CD8+ T cell dysfunction has also been described in chronic FV infection of mice^9, 10^.

FV infection is a well-established mouse model to study mechanisms of immunity against retroviruses. It has been shown that exhausted CD8+ T cells cannot control viral replication in chronic FV infection, but viral loads are still under control of the immune system^11^. Thus, chronically infected mice can maintain low viral set points and usually don’t progress to leukemia. During a series of experiments Hasenkrug *et al*. showed that the cell population responsible for control of chronic FV was CD4+ T lymphocytes and that this control was lost in CD4 depleted animals^11^. These results suggested that CD4+ T cells might mediate direct antiviral effects during chronic FV infection. However, the question about possible *in vivo* targets for cytotoxic CD4+ T cells remained unanswered. Potential targets should to be virus infected and express MHC class II. Interestingly, we recently demonstrated that FV-infected B cells and myeloid cells escape from CD8+ T cell-mediated killing during the acute phase of infection and subsequently form the viral reservoir during chronic FV infection^12^. These cells may therefore be perfect targets for CD4+ T cells since they express viral antigens and are MHC class II positive.

The idea that CD4+ T cells may play a significant role in mediating direct anti-viral effects in chronic viral infections generated attention of scientists in the last decade. It has been shown in both human^13^ and mouse models^14^ that CD4+ T cells might exert direct antiviral activities in the setting of low level viremia. The evidence of CD8+ T cell exhaustion with simultaneous direct anti-viral CD4+ T cell effects in the chronic phase of infection led us to hypothesize that CD4+ T cells may have cytotoxic activity during chronic FV infection. Indeed an FV-specific CD4+ T cell clone that could kill FV-infected target cells *in vitro* was described^15^. However, this clone was not obtained from chronically infected mice, but from an animal that was challenged with the FV-transformed tumor cell line FBL-3. In addition, no CD4+ T cell cytotoxicity was found during acute FV infection^16, 17^. Therefore, the mechanisms of CD4+ T cell-mediated virus control during the chronic phase of FV infection remained unclear.

The cytotoxicity of CD4+ T cells has been described and recognized in cancer models for quite some time^18^. However, the mechanisms of direct CD4+ T cell-mediated killing are still not clear due to the lack of MHC class II on most cells from solid cancers^19^. The first evidence supporting CD4+ T cell dependent rejection of cancer cells came from melanoma models^20^. In those studies CD4+ T cells were shown to secrete effector cytokines^21^, recruit other cell populations^22^, offer help for generating memory CD8+ T cells^23^ and induce direct cytotoxic killing of tumor cells via granzyme-dependent mechanisms^24^.

Here we carefully characterized the activation and functional properties of effector CD4+ T cells during the chronic phase of FV infection. Importantly, we demonstrate CD4+ T cell-mediated killing of FV-labeled target cells with an *in vivo* MHC class II CTL assay. Finally, we identified the Fas/FasL pathway of apoptosis to mediate the CD4+ T cell cytotoxicity in the chronic phase of FV infection.

## Results

### Kinetics of viral load during FV infection

**Figure 1:**
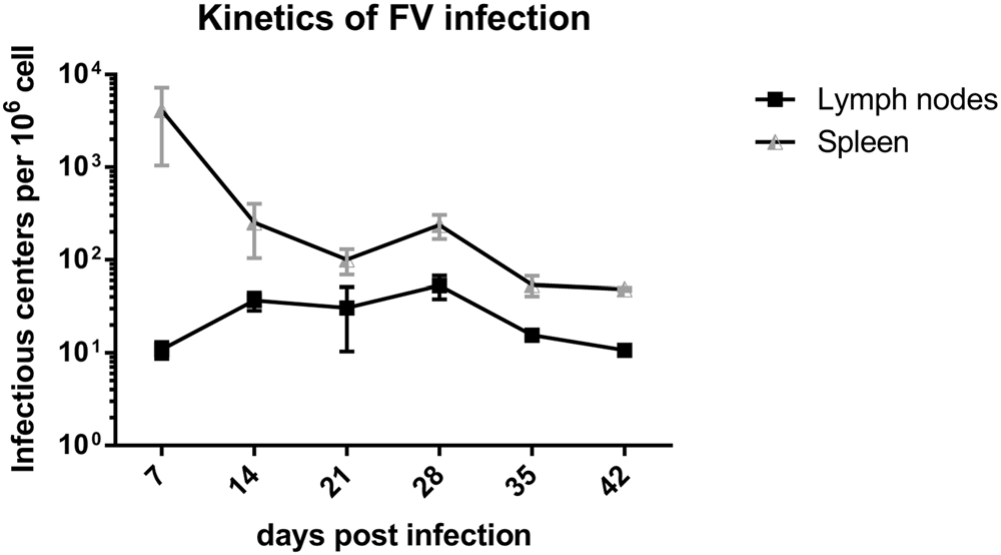
Kinetics of FV infection. Viral loads of FV-infected C57BL/6 mice were analyzed in spleens and lymph nodes using an infectious center assay. At least six mice per group were analyzed. Data were pooled from two independent experiments.

The main organs for FV replication during the acute phase of infection are bone marrow and spleen^25^. The kinetics of viral loads in these organs was already shown in previous publications^10, 26^. However during chronic FV infection the main viral reservoir was found in the lymph nodes and spleen^25^. The kinetics of viral infection in the spleens and lymph nodes of FV-infected C57BL/6 mice 7 days post infection (7 dpi) to 42 dpi are shown in Fig. 1. To reproducibly establish chronic infection in leukemia-resistant C57Bl/6 mice they have to be infected with high doses of FV complex plus additional inoculation of F-MuLV helper virus to facilitate virus replication *in vivo*. Viral loads declined in spleens over this time period, but were still detectable at 4 dpi (Fig. 1). In contrast, no virus can be found anymore in the bone marrow of most FV-infected mice at this time point^25^. FV-infected cells were detected in lymph nodes during the acute phase of infection, although the numbers were rather low (Fig. 1). Over time the viral loads in lymph nodes remained quite stable and virus could still be detected in all infected mice at 42 dpi. These results confirm the presence of chronic FV in spleens and lymph nodes, which suggest that immune mechanisms of control must be maintained in those two organs to keep virus replication in check. Therefore, all subsequent studies concentrated on spleens and lymph nodes cells.

### Activation and proliferation of FV-specific CD4+ T cells during the chronic phase of FV infection

**Figure 2:**
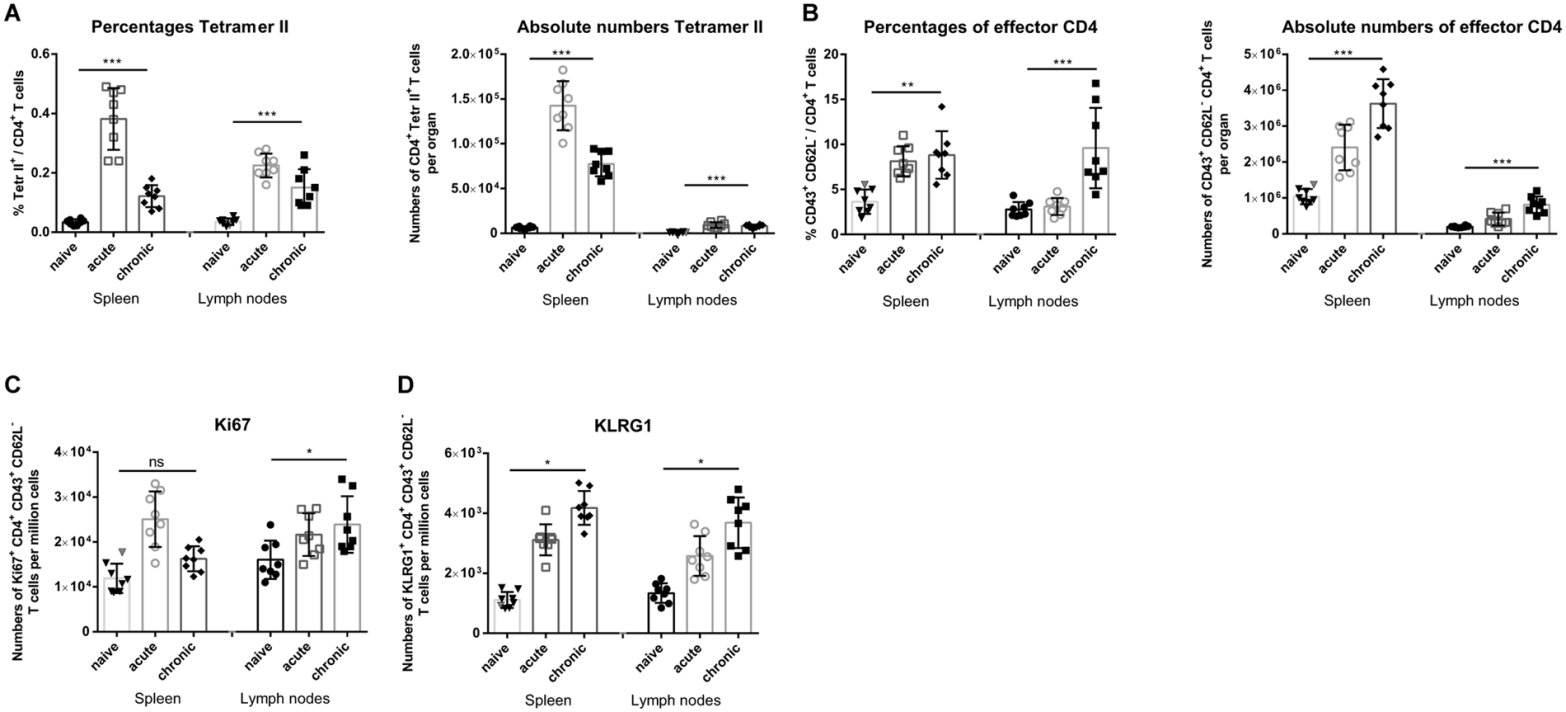
CD4+ T cell response during chronic FV infection. C57BL/6 mice were chronically infected with FV (6 weeks post infection). (**A**) Percentages and absolute numbers of CD4+ Tetr II+ T cells reactive with I-Ab MHC class II tetramers specific for the FV H19-Env epitope. (**B**) Percentages and absolute numbers of CD4+ T cells showing the activation profile CD43+ and CD62L−. (**C**) Numbers per million cells of effector CD4+ T cells expressing proliferation marker Ki67. (**D**) Numbers per million cells of effector CD4+ T cells expressing the differentiation marker KLRG1. Each dot represents an individual mouse. Mean values are indicated by a line. Statistically significant differences between the groups were determined by the unpaired t test: *p < 0.05; **p < 0.005; ***p < 0.0001, ns, not significant. Data were pooled from two independent experiments.

To determine whether virus-specific CD4+ T cells remained detectable during chronic FV infection, spleen and lymph node cells were analyzed using MHC II tetramer (Tet II) (constructed for the FV H19-Env epitope). The overall CD4+ T cell population responding to FV infection was previously characterized as CD43+CD62L− cells^16^. The CD4+ T cells expanded in response to acute infection (Fig. 2) and remained detectable during chronic infection in both spleens and lymph nodes (Fig. 2A and B).

Interestingly, the frequency of proliferating (Ki67+) and highly differentiated (KLRG1+) CD4+ T cells was even higher in chronic FV infection than in acute FV infection, with the exception of Ki67+ cells in the spleen (Fig. 2C and D). These data indicate an ongoing CD4+ T cell response during chronic FV infection. However, numbers of Tet II+ CD4+ T cells were quite low during chronic infection and Tet II staining cannot be combined with intracellular staining, which is why all functional analyses were performed with activated CD43+CD62L− CD4+ T cells.

### CD4+ T cells produce no granzymes, but express Fas Ligand during chronic FV infection

**Figure 3:**
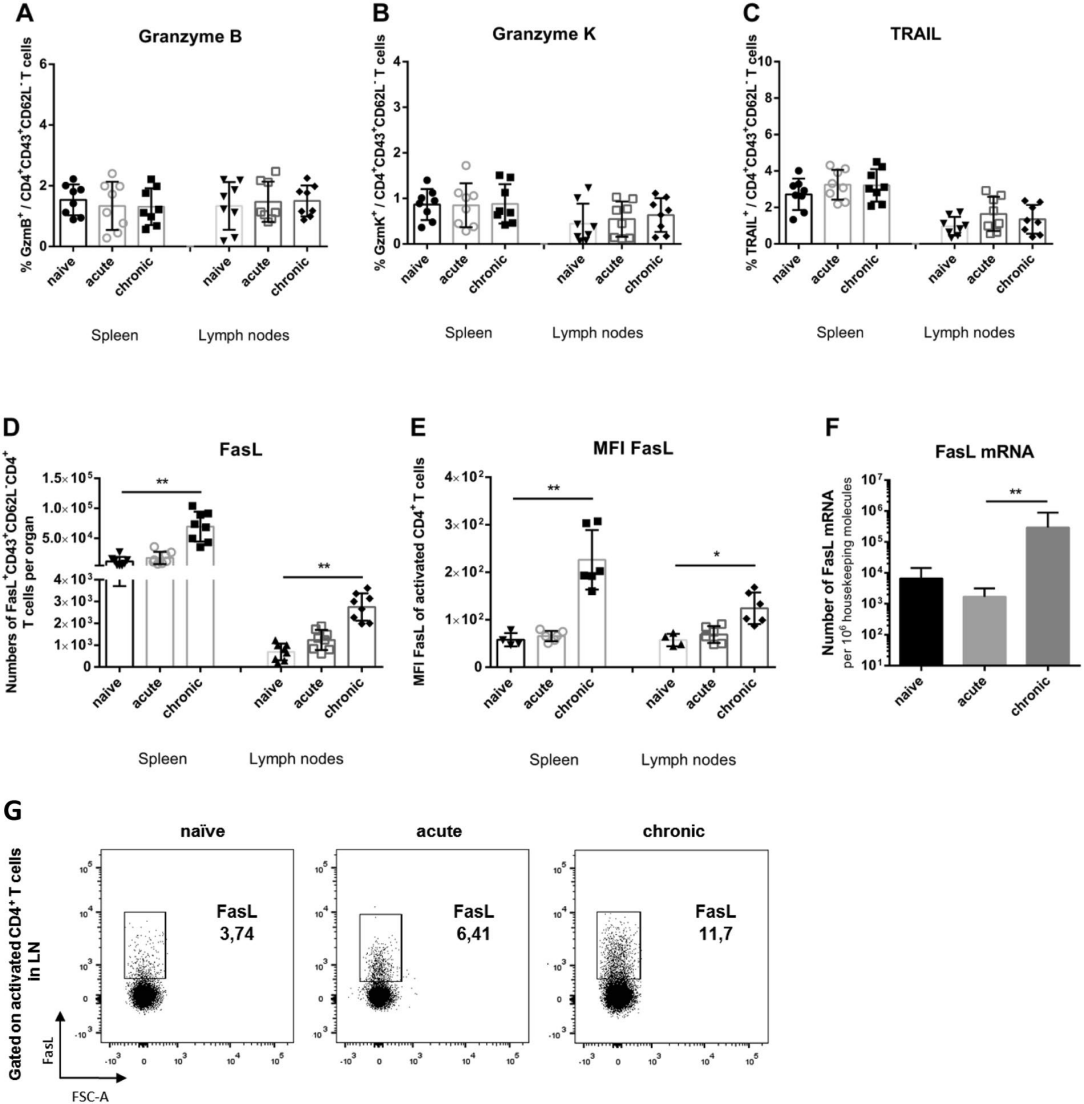
Expression of cytotoxic molecules in effector CD4+ T cells from chronically FV infected mice. Flow cytometry was used to detect intracellular granzymes, or surface Fas ligand in activated effector CD4+ T cells (CD43+CD62L−). (**A**) Percentages of effector CD4+ T cells expressing granzyme B. (**B**) Percentages of effector CD4+ T cells expressing granzyme K. (**C**) Percentages of effector CD4+ T cells expressing TRAIL after restimulation with αCD3 and αCD28 antibodies *in vitro*. (**D**) Absolute numbers of effector CD4+ T cells expressing FasL after restimulation with αCD3 and αCD28 antibodies *in vitro*. (**E**) FasL expression is presented as in D), except that data are expressed as MFI. (**F**) mRNA levels for FasL in effector CD4+ T cells isolated from FV infected mice. β-actin was used as an internal standard. (**G**) Representative dot plots of FasL expression on activated CD4+ T cells from different phases of FV infection. Each dot represents an individual mouse. Mean values are indicated by a line. Statistically significant differences between the groups were determined by the unpaired t test: *p < 0.05; **p < 0.005. Data were pooled from at least two independent experiments.

Activated CD4+ T cells with direct anti-viral activity may express cytotoxic molecules during chronic FV infection. We investigated that possibility by staining for different granzymes or the ligand for the death receptors Fas or TRAIL receptor. This analysis was performed by gating on CD4+ T cells with an effector phenotype (CD43+CD62L−). Interestingly, the population of effector CD4+ T cells from acutely and chronically infected mice did not show enhanced percentages of cells expressing granzyme B (Fig. 3A), granzyme K (Fig. 3B) or granzyme A (data not shown) compared to naïve controls. This suggested that activated CD4+ T cells may use a different pathway than the exocytosis pathway to mediate their anti-viral effects.

Tumor necrosis factor-related apoptosis-inducing ligand (TRAIL) is a member of the tumor necrosis factor family that initiates apoptosis of cells through engagement to its receptor^27^. TRAIL was shown to play an important role in T cell-mediated killing of both tumor cells^28^ and virus infected cells^29, 30^. However, we couldn’t detect any significant differences in TRAIL expression on activated CD4+ T cells between chronically infected mice and naïve or acutely infected animals (Fig. 3C). Next, the expression of Fas Ligand (FasL) on effector CD4+ T cells was examined. Interaction of Fas and FasL results in apoptotic cell death^31^ previously described to be involved in the killing of virus infected cells^32^. In both spleens and lymph nodes were found significantly increased frequencies and absolute numbers of effector CD4+ T cells expressing FasL (Fig. 3D and E). Representative dot plots of FasL expression on activated CD4+ T cells from different phases of FV infection are shown in Fig. 3G. To confirm these results, we also compared mRNA expression levels for FasL in sorted CD43+CD62L−CD4+ T cells. We detected significantly more mRNA FasL molecules in cells from chronically infected mice compared to the other two groups (Fig. 3F). These results suggested that effector CD4+ T cells might mediate their anti-viral effects during chronic FV infection via the Fas/FasL cytotoxicity pathway.

### *In vivo* cytotoxicity assay allows the detection of FV-specific CD4+ T cell mediated killing of target cells

**Figure 4:**
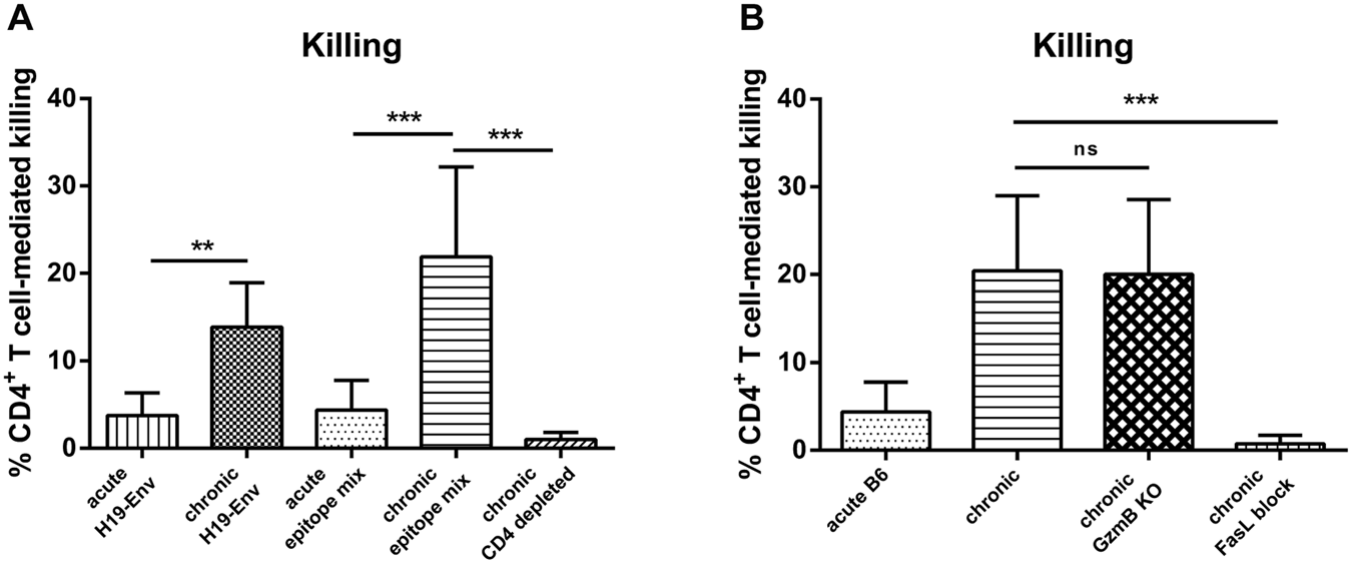
*In vivo* cytotoxicity of CD4+ T cells during chronic FV infection. Lymph node cells and spleen cells from naïve mice were loaded with CD4+ T cell FV specific epitope peptides and labeled with CFSE dye. Unloaded cells were stained with cell trace violet. Peptide loaded and unloaded cells were injected intravenously in a 1:1 ratio into naïve and FV-infected mice and tracked by flow cytometry 20 hours after injection. The figure shows the percentage of the target cell killing in the lymph nodes. (**A**) Cells from naïve mice were loaded either with H2-A^b^-restricted F-MulV H19 envelope epitope alone (H19-Env) or with nine additional epitope peptides spanning the coding regions gag, pol, and env (epitope mix), which were described by R.J. Messer *et al*.^33^ and injected intravenously into naïve, acutely FV-infected (n = 8), chronically FV-infected (n = 12), chronically FV-infected CD4^+^ T cell depleted mice (n = 4). (**B**) Cells from naïve mice were loaded with the peptide epitope mixture. Peptide loaded and unloaded cells were injected intravenously into naïve, acutely FV-infected (n = 8), chronically FV-infected (n = 12), chronically FV-infected GzmB KO mice (n = 8), chronically FV-infected mice, receiving a FasL blocking antibody (n = 4). Mice receiving an isotype control antibody did not show any difference to the non-treated control group. Mean values are indicated by a line. Statistically significant differences between the groups were determined by the unpaired t test: **p < 0.005; ***p < 0.0001, ns, not significant. Data were pooled from two independent experiments.

We previously developed an MHC class II-restricted *in vivo* cytotoxicity assay to detect FV-specific killing of target cells loaded with the immunodominant FV CD4+ T cell epitope H19-Env. However, if mice were infected with FV no specific killing was detected during acute infection^16^. In contrast, in chronically infected mice the killing of H19-Env peptide-loaded target cells in the lymph nodes was at around 15% (Fig. 4A). This was significantly higher in comparison with acutely infected mice. To improve the breadth of this *in vivo* killing assay we took advantage of the recent identification of 9 other FV-specific CD4+ T cell epitopes in the Gag, Pol and Env proteins^33^. To measure the simultaneous killing of all CD4+ T cells with these different specificities we used a mixture of 10 epitope peptides for labeling our target cells.

Even with this epitope mixture no significant FV-specific CD4+ T cell killing was detected during acute infection (Fig. 4A). However, during chronic FV infection CD4+ T cell mediated killing increased to a mean of over 20%, with some animals showing up to 35% target cell killing (Fig. 4A). In order to prove that this killing of FV epitope peptide labeled target cell was mediated by CD4+ T cells we depleted CD4+ T cell in chronically infected mice and performed the *in vivo* CTL assay in these animals. Indeed, no killing of target cells was observed when CD4+ T cells were absent (Fig. 4A).

### CD4+ T cell-mediated cytotoxicity in chronically FV-infected granzyme B knockout mice, but not in wild type mice receiving FasL blocking antibodies

Our data in Fig. 3 suggested that the CD4+ T cell-mediated cytotoxicity was FasL-dependent. To verify this, the *in vivo* cytotoxicity assay was performed in chronically infected granzyme B knockout mice and in mice with antibody-mediated blockade of the Fas/FasL pathway. Killing in granzyme B knockout mice was equivalent to that observed in wild type mice (Fig. 4B) confirming that the cytotoxicity by CD4+ T cells was not dependent on the exocytosis pathway.

In contrast, CD4+ T cell-mediated killing was completely abrogated in chronically infected mice following injection of FasL-blocking antibody (Fig. 4B). These results indicated that the CD4+ T cell cytotoxicity during chronic FV infection was mediated by the Fas/FasL pathway.

**Figure 5:**
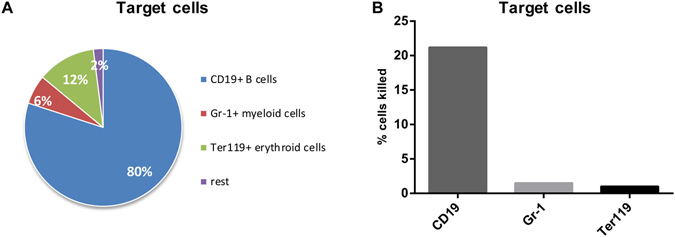
CD4+ T cell cytotoxicity against MHC II+ target cells. Target cells for the CD4+ T cell cytotoxicity assay were obtained from lymph nodes and spleens of naïve mice, loaded with epitope peptides and injected into FV-infected recipient mice. The target cell population was depleted for T cells to increase the frequency of possible MHC II+ targets. (**A**) Distribution of different target cell populations in the lymph nodes of naïve recipient mice. (**B**) Frequencies of killed cells indicated that CD19+ B cells were the main targets of cytotoxic CD4+ T cells. The data were pooled from two independent experiments with similar results.

Next, we wanted to determine the target cell populations that were killed by the FasL-expressing CD4+ T cells. First, the distribution of target cells in lymph nodes from uninfected recipient mice was analyzed. Figure 5A shows that CD19+ B cells form the major target cell population after transfer, but also Ter119+ erythroid cells and Gr-1+ myeloid cells were detectable. Since T cells were depleted prior to injection of the target cells in order to enrich MHC class II-positive cells, virtually no CD3+ T cells were found. Then, the percentage of target cell killing in chronically FV-infected mice was determined for the different subpopulations. Only few Ter119+ erythroid precursor cells or Gr-1+ myeloid cells (less than 2%) were killed, whereas around 20% of the CD19+ B cells labeled with FV epitope peptides were eliminated *in vivo* (Fig. 5B). Thus, B cell killing accounted for nearly all the cytotoxic activity of CD4+ T cells that we detected in chronically FV-infected mice. This is in line with what we know about the control of persistent FV, since B cells are the main reservoir of the virus during chronicity^11^.

### CD134 signaling only elicits activation of CD4+ T cells, while αCD137 antibody treatment induced the exocytosis pathway

Our previous data indicates that chronic FV infection is controlled by cytotoxic CD4+ T cells that mediate killing via the Fas/FasL pathway. However, these cytotoxic cells were only able to restrict virus replication to a constant low level and prevent onset of virus-induced pathology^11^. Cytotoxic CD4+ T cells that use the exocytosis pathway for killing virus-infected targets might be more efficient in reducing chronic viral loads, which might be an interesting therapeutic concept. CD134 and CD137 are known as co-stimulatory molecules, which can induce the exocytosis pathway of cytotoxicity in CD4+ T cells^34, 35^. We therefore used agonistic antibodies against these molecules, to augment cytotoxicity of CD4+ T cells during chronic FV infection. Chronically FV-infected mice were treated 6 times with either αCD134 or αCD137 antibody. Using flow cytometry we characterized the result of treatment on effector phenotype CD4+ T cells, with a focus on activation, proliferation, differentiation, and cytotoxic molecules. One of those molecules was Eomesodermin (Eomes), a T-box transcription factor, which has been described as master regulator of the exocytosis pathway in cytotoxic cells^36^.

**Figure 6:**
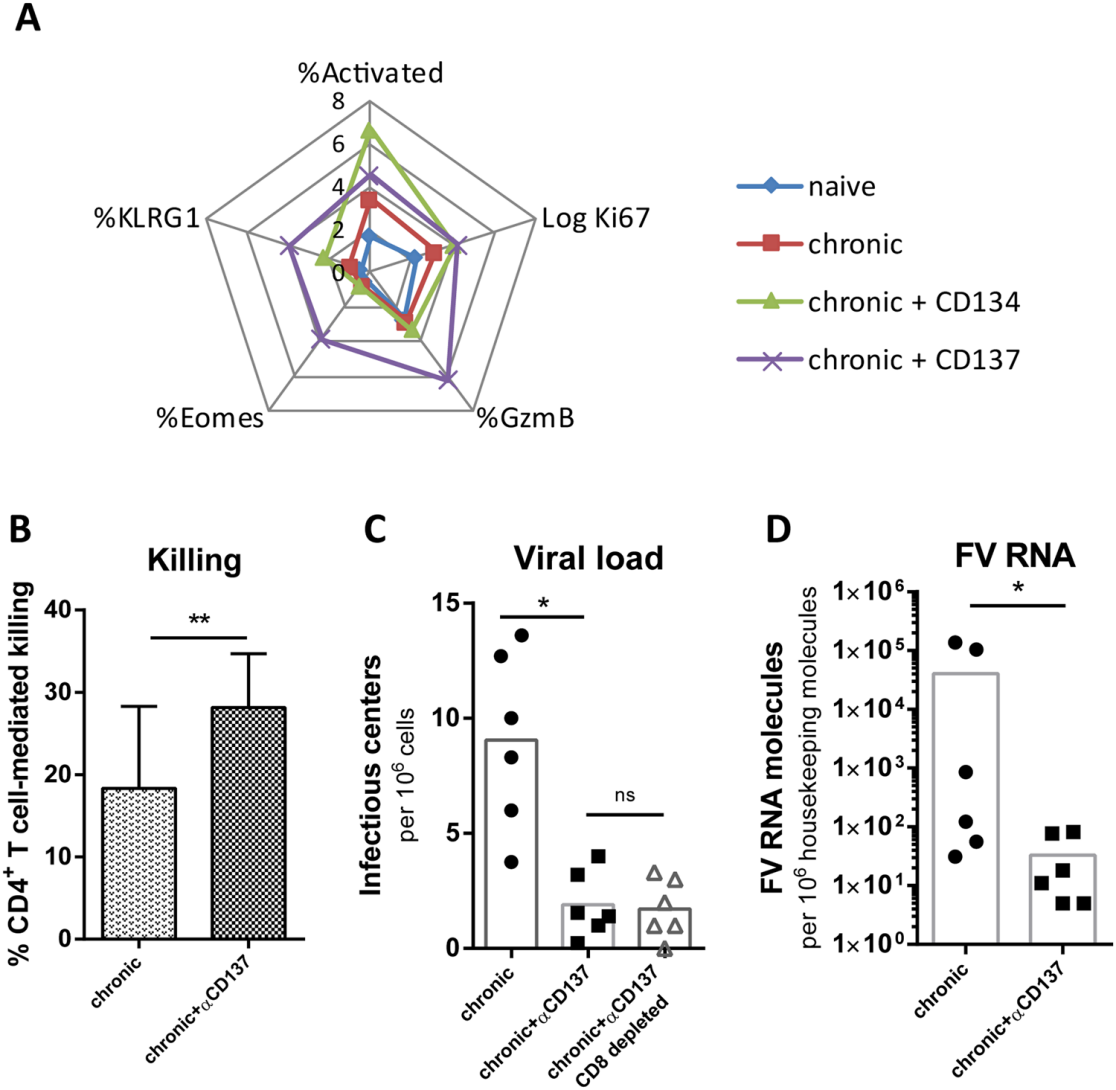
Effector cell profile of CD4+ T cells following costimulatory therapy. To augment CD4+ T cell cytotoxicity FV-infected mice were treated with αCD134 or αCD137 antibodies as described in Materials and Methods. (**A**) Spider plot analysis showing the expression levels of CD4+ T cell activation, differentiation and cytotoxic markers after antibody therapy. αCD134 antibody treatment only enhanced activation of CD4+ T cells, but only αCD137 antibodies induce expression of molecules associated with a cytotoxic program. (**B**) The percentage of the target cell killing in the lymph nodes of FV-infected mice following αCD137 antibody treatment. Mice receiving an isotype control antibody did not show any difference to the non-treated control group. (**C**) Viral loads in FV-infected mice after CD137 treatment. (**D**) FV RNA levels after CD137 treatment.Statistically significant differences between the groups were determined by the unpaired t test: *p < 0.05; **p < 0.005; ***ns, not significant.

αCD134-treated mice had higher frequencies of activated CD4+ T cells than untreated controls, but no significant induction of the exocytosis pathway was found (Fig. 6A). In contrast, αCD137 treatment did not result in strong activation of CD4+ T cells, but induced expression of Eomes and granzyme B, indicating that CD137 signaling induced the exocytosis pathway in CD4+ T cells. Therefore αCD137 antibodies were used for immunotherapy experiments.

An *in vivo* cytotoxicity assay revealed that αCD137 treatment indeed resulted in a significant improvement of target cells killing in the lymph nodes of chronically FV-infected mice (Fig. 6B). The improved CD4+ T cell cytotoxicity was associated with significantly reduced chronic viral loads in mice after receiving αCD137 antibodies measured by an infectious center assay and by quantitative RNA PCR. This effect of αCD137 immunotherapy was mainly mediated by CD4+ T cells, as CD8-depletion did not abrogate the therapeutic efficacy (Fig. 6C and D).

Taken together, the data indicates that αCD137 therapy can induce the exocytosis pathway in cytotoxic CD4+ T cells during a chronic retroviral infection and might therefore be an interesting therapeutic approach to treat chronic viral infections.

## Discussion

CD4+ T cells play an important role as helper cells in many acute infections. This cell population is also facilitating neutralizing antibodies (NAb) responses, which are necessary for recovery from acute FV infection^37^. However, the role of NAb during the chronic phase of FV infection has not been clearly defined. The only available data from an earlier study describes no correlation between the titers of NAb and disease relapse in chronic FV infection after an experimental CD4+ T cell depletion^11^. This may indicate that NAb do not play a major role in the control of chronic FV infection.

During chronic phase of infections when CD8+ T cells often become functionally exhausted, the CD4+ T cells appear to take over some of the direct antiviral activities of CD8+ T cells. CD4+ T cells are known for their phenotypic plasticity, which might allow them to perform multiple functions during an ongoing infection. During acute FV infection we were unable to detect CD4+ T cell cytotoxicity in either the current or in previous studies^16^. In fact, we showed that the cytotoxicity of CD4+ T cells was under the control of both regulatory T cells and CD8+ T cells. Thus, during acute FV infection, CD4+ T cells were only cytotoxic when both Tregs and CD8+ T cells were depleted. The molecular mechanisms are not very well understood, but co-stimulatory receptors on effector CD4+ T cells, like CD134 or CD137 might play a role in this regulation^35^. Cytotoxicity of CD4+ T cells can be associated with immunopathology during acute infections and it might therefore be biologically important to suppress this response. Indeed, a number of recent studies showed that strongly augmented CD4+ T cells responses can result in the death of mice during an acute viral infection^38^. However, whether CD4+ T cell cytotoxicity was involved in this immunopathology was not investigated. As long as cytotoxic CD8+ T cells are present and mediate killing of infected target cells, cytotoxic CD4+ T cells are probably not needed. This situation changes during chronic infection when CD8+ T cells have diminished cytotoxic potential. It is therefore not surprising that cytotoxic CD4+ T cells have mainly been found in chronic infections, including HIV^39–41^. CD4+ T cells can also become dysfunctional when a persistent infection develops which has been demonstrated in several virus infections^42^, including HIV infection of humans^43^. In fact, we also showed the development of CD4+ T cell exhaustion during the late phase of acute FV infection, indicating the FV infection is no different than other chronic infections in this aspect. The CD4+ T cell dysfunction that we described affected the ability of FV-specific CD4+ cells to produce cytokines (IFNγ and IL2)^44^. In our current work we demonstrate that the Fas/FasL pathway of cytotoxicity is not affected by the mechanisms of CD4+ T cell exhaustion. This is in line with our previous findings in studies on CD8+ T cells which showed that regulatory T cells suppress the exocytosis pathway in FV-infected mice, but allow CD8+ T cell to kill infected targets via the Fas/FasL pathway^45^. In HIV infection it was shown that CD4+ T cells can kill HIV-infected target cells *in vitro* ^46^ and they even work cooperatively with HIV-specific CD8+ T cells to control HIV replication in infected patients^40^. Historically there where several lines of evidence in the FV model for the activity of cytotoxic CD4+ T cells during chronic infection, but until recently it was technically not possible to analyze this response. We were finally able to do so by developing a modified *in vivo* cytotoxicity assay for CD4+ T cells.

We could also answer the question what target cells are actually affected by cytotoxic CD4+ T cells. The main cell population that is infected during acute FV infection are Ter119+ erythroid precursor cells^47^. However, these cells are efficiently eliminated by CD8+ T cells during acute FV infection^32^ and they do not express MHC class II. FV also infects B cells and Myeloid-derived cells and some of these infected cells escape from CD8+ T cell-mediated killing during acute infection^12^ and subsequently form the viral reservoir for chronic infection^48^. Since they express MHC class II they are potential targets for CD4+ T cell-mediated killing. Indeed, we show with our *in vivo* CTL assay that FV-labeled CD19+ B cells were eliminated in chronically infected mice.

We also analyzed the pathway that the CD4+ T cells utilized for their cytotoxic activity. Interestingly, the CD4+ T cells did not express granzymes during chronic FV infection and the exocytosis pathway was not involved in cytotoxicity. In contrast, CD4+ T cells with effector phenotype expressed FasL on their surface and mediated target cell killing by Fas/FasL interaction.

Our current results demonstrate that effector CD4+ T cells mediate potent cytotoxic activity that can keep virus in check, but they are not able to eliminate all virus infected target cells. Induction of CD4+ T cell cytotoxicity in chronic FV infection caused no immunopathology as previously described for some other virus infections^38^. As this cytotoxic activity is under the control of Tregs, CD4+ T cells only use the Fas/FasL pathway to induce killing of infected target cells. Not surprisingly, Fas/FasL knockout animals lost control over chronic FV infection^10^. In the current study we show that CD4+ T cells can reveal their cytotoxicity during the chronic phase of FV infection via the Fas/FasL pathway. The data indicate that cytotoxicity by CD4+ T cells is a critical factor for the immune control of chronic retroviral infection and that this cytotoxicity can be augmented by immunotherapy to achieve superior suppression of chronic virus.

## Methods

### Mice

Inbread C57BL/6 and GzmB KO mice were maintained under pathogen-free conditions. Experiments were done using mice on the C57BL/6 background that are resistant to FV-induced leukemia. All mice were females of 8 to 16 weeks of age at the beginning of the experiments.

### Ethics statement

Animal experiments were performed in strict accordance with the German regulations of the Society for Laboratory Animal Science (GV-SOLAS) and the European Health Law of the Federation of Laboratory Animal Science Associations (FELASA). The protocol was approved by the North Rhine-Westphalia State Agency for Nature, Environment and Consumer Protection (LANUV) (Permit number: G 1518/15). All efforts were made to minimize suffering.

### Virus and viral infection

The FV stock used in the experiments was a FV complex containing B-tropic Friend murine leukemia helper virus and polycythemia-inducing spleen focus-forming virus. The stock was prepared as a 15% spleen cell homogenate from BALB/c mice infected 14 days previously with 3,000 spleen focus-forming units (SFFU). Mice were infected intravenously with 20,000 SFFU for acute infection. For the development of chronic infection, additional 100,000 FFU of Friend murine leukemia helper virus (F-MuLV) were added. The stock was lactate dehydrogenase virus (LDV) - free.

### Flow cytometry

Cell surface and intracellular stainings were performed using antibodies as follows: CD4 (RM 4–5; BioLegend), CD8 (53–6.7; eBioscience), CD43 (1B11; BioLegend), CD62L (MEL-14; BioLegend), CD11b (M1/70; BioLegend), KLRG1 (2F1/KLRG1; BioLegend), Ki67 (16A8; BioLegend), FasL (MFL3; BD Biosciences), TRAIL (N2B2; eBioscience), Foxp3 (FJK-16s; eBioscience), GzmB (GB12; ThermoFisher Scientific), GzmA (GzA-368.5; eBioscience) and Gzm K (Orb102688; Biorbyt). Dead cells were excluded by fixable viability dye (eBioscience) staining. For FasL staining, lymphocytes were isolated and restimulated with anti-CD3 (145-2C11, eBioscience) and anti-CD28 (37.51, eBioscience) antibodies for 5 hours at 37 °C. BD Cytofix/Cytoperm Fixation/Permeabilization kit was used for intracellular staining following the manufacturer’s instructions. Data were acquired on an LSR II flow cytometer (BD Biosciences). Analyses were done using FlowJo 5.0 software (Tree Star Inc., Ashland, OR).

### Tetramers and tetramer staining

MHC class-II tetramers loaded with I-A b-restricted FV-specific CD4+ T-cell epitope (H19-Env; EPLTSLTPRCNTAWNRLKL) were obtained from the National Institutes of Health Tetramer Facility (Atlanta). Tetramers were used for detection of I-A b FV Env-specific CD4+ T cells. Spleens and lymph nodes cells were incubated with APC-labelled I-A b tetramers for 2 h at 37 °C and later stained with surface antibodies at room temperature.

### Infectious center assays

Single cell suspensions from infected mouse lymph nodes and spleens were plated onto susceptible *Mus dunni* cells and incubated at 37 °C and 5% CO_2_ for 3 days. Cells were then fixed with 95% ethanol, stained with F-MuLV envelope-specific mAb 720, and then viral foci developed with peroxidase-conjugated anti-mouse IgG and substrate.

### Quantitative PCR

Total RNA was extracted from CD43+CD62L−CD4+ T cells by using the innuPREP RNA Mini Kit (Analytik Jena, Jena, Germany) according to the manufacturer’s instructions. The resulting RNA was used for amplification in the Rotor-Gene Q system (Qiagen, Hilden, Germany) by using Power SYBR Green RNA-to-C_T_ 1-Step Kit (Applied Biosystems, Thermo Fisher Scientific, Waltham, USA).

### Cell isolation

CD4+ T cells from spleens and lymph nodes were isolated with MagniSort^®^ Mouse CD4 T cell Negative Selection Kit (eBioscience) according to manufacturer’s instruction. Purity of the isolated CD4+ T-cell population was higher than 85%.

### Monoclonal antibodies and Fas/FasL interaction blockade

Low endotoxin azide-free purified anti-mouse CD178 (FasL) antibody (clone MFL3) for *in vivo* use was purchased from BioLegend (San Diego, CA). To block Fas/FasL interactions, 250 µg anti-mouse CD178 antibody was diluted in 300 µl PBS and injected intraperitoneally once 24 hours before the cytotoxicity assay.

### CD134/CD137 agonist treatment

The anti-CD134 (OX-86) and anti-CD137 (LOB 12.3) used *in vivo* were produced by BioXCell. Dosing per injection was 100 μg administered i.p. 6 times every second day starting at 43 dpi post infection. In some mice also CD8+ T cells were depleted. They received i.p. 0.5 ml supernatant fluid obtained from hybridoma cell line 169.4 producing CD8a-specific mAb. Depletion was started at 42 dpi and carried out every second day until mice were killed. The treatment depleted >95% of the CD8+ T cells.

### *In vivo* cytotoxicity (*in vivo* CTL) assay

Cell suspension from lymph nodes and spleens were incubated for 1.5 hours at 37 °C with the MHC class II-restricted peptides mixture, which is recognized by CD4+ T cells. Cells were stained with CellTrace^TM^ CFSE dye (ThermoFisher Scientific). MHC class II-restricted peptides recognized by CD4+ T cells were described previously^33, 49^ and purchased from Peptides&elephants GmbH (Potsdam, Germany). Unloaded cells had been stained with CellTrace^TM^ Violet (ThermoFisher Scientific). Peptide loaded and unloaded cells were suspended in PBS in a 1:1 ratio. Cell suspension containing 3.0 × 10^6^ cells of each population was injected intravenously into each mouse. 20 hours later, lymph nodes and spleens have been harvested. The ratio of live CFSE+ target cells loaded with peptides recognized by CD4+ versus Violet+ unloaded target cells have been calculated normalized to the ratio in naive animals.

### Statistics

Statistical analyses were performed using the nonparametric Student *t* test and graphical presentation was performed using Graph Pad Prism version 6.

### Data Availability

The datasets generated and analyzed during the current study are available in the Sciebo repository: https://uni-duisburg-essen.sciebo.de/index.php/s/mobj2gz6vrXxK8E.
